# The RNA Binding Protein Csx1 Promotes Sexual Differentiation in *Schizosaccharomyces pombe*

**DOI:** 10.1371/journal.pone.0030067

**Published:** 2012-01-11

**Authors:** Ana M. Matia-Gonzalez, Jael Sotelo, Miguel A. Rodriguez-Gabriel

**Affiliations:** Centro de Biología Molecular “Severo Ochoa,” Universidad Autónoma de Madrid (UAM), Consejo Superior de Investigaciones Científicas (CSIC), Madrid, Spain; University of Cambridge, United Kingdom

## Abstract

Sexual differentiation is a highly regulated process in the fission yeast *Schizosaccharomyces pombe* and is triggered by nutrient depletion, mainly nitrogen source.

One of the key regulatory proteins in fission yeast sexual differentiation is the transcription factor Ste11. Ste11 regulates the transcription of many genes required for the initial steps of conjugation and meiosis, and its deficiency leads to sterility. Ste11 activity is mainly regulated at two levels: phosphorylation and abundance of its mRNA.

Csx1 is an RNA binding protein that we have previously described to bind and regulate the turnover rate of the mRNA encoding the transcription factor Atf1 in the presence of oxidative stress.

We have observed that Csx1-deficient cells have defects in sexual differentiation and are partially sterile. We investigated how Csx1 is regulating this process in *S. pombe*. Csx1 associates with s*te11^+^* mRNA and cells lacking Csx1 are sterile with a reduced amount of s*te11^+^* mRNA. Overexpression of s*te11^+^* mRNA completely rescues the mating deficiencies of *csx1*Δ cells.

Here, we present a novel mechanism of s*te11^+^* mRNA positive regulation through the activity of Csx1, an RNA binding protein that also have key functions in the response to oxidative stress in fission yeast. This finding opens interesting question about the possible coordination of sexual differentiation and oxidative stress response in eukaryotes and the role of RNA binding proteins in the adaptation to environmental signals.

## Introduction

In the fission yeast *S. pombe*, haploid homothallic (*h^90^*) and heterothallic (*h^+^* or *h^−^*) strains reproduce by mitosis and are divided by medial fission under standard growth conditions. Under nitrogen starvation conditions, cells with opposite mating type exchange mating pheromones that promote cell fusion, inducing conjugation and forming diploid zygotes. Diploids then undergo meiosis and sporulation, producing four haploid spores (tetrad) that, under adequate conditions, would germinate finishing the mating cycle [1].

The transcription factor Ste11 is a key factor in the sexual differentiation process in *S. pombe* [2]. Ste11 participates in the transcriptional induction of many genes involved in the mating process, including the key meiotic regulator Mei2, and cells deficient in Ste11 are profoundly sterile [2], [3], [4], [5]. The activity of Ste11 is regulated at several levels [1]. First, Ste11 protein is phosphorylated by several kinases including Pat1, Spk1 and Cdc2 [6], [7], [8], [9], and second, Ste11 mRNA abundance is regulated by Atf1, Rst2, Pcr1 and Prr1 transcription factors [10], [11], [12], [13], [14], [15] and Msa1 and Msa2/Nrd1 RNA binding proteins [16], [17], [18].

Most of these processes are controlled by signaling systems that detect nutritional changes in the environment and trigger the transition mitosis-meiosis through the conjugation/meiosis pathway. Thus, sexual differentiation in *S. pombe* is regulated by several signaling pathways, like the cAMP pathway (PKA), the MAPK pheromone signaling pathway (Spk1), the TOR pathway, and the MAPK stress-responsive Sty1/Spc1 pathway. PKA negatively regulates mating, while the MAPKs Spk1 and Spc1/Sty1 positively regulate the mating process. TOR kinases, Tor1 and Tor2, exert positive and negative effects on mating, respectively [1], [11], [13], [19], [20].

One of the mechanisms of Ste11 regulation is through the activity of the Spc1/Sty1 MAPK pathway [13]. Upon nitrogen starvation, Atf1, a transcription factor regulated by Spc1/Sty1, activates Ste11 transcription and, therefore, mating capacity [10], [13], [14]. Cells deficient in Spc1/Sty1 or Atf1 are not capable of arresting cell cycle in G1 upon nitrogen starvation and are, therefore, sterile.

*atf1^+^* mRNA levels, under certain stress conditions, like hydrogen peroxide treatment, are regulated by the activity of Csx1, an RNA binding protein with 3 RNA recognition motifs. Csx1 phosphorylation depends on Spc1/Sty1 activity, although the functional role of this phosphorylation remains unclear [21].

We have noticed that Csx1-deficient cells may also have defects in mating. In this report we have examined the role of Csx1 RNA binding protein in the sexual differentiation process in *S. pombe*.

## Results

### 1. Function of Csx1 in sexual differentiation

During the construction of strains containing different Csx1 alleles, we noticed that the mating efficiency of heterothallic Csx1-deficient strains was lower than wild type strains, indicating a possible role of Csx1 in sexual differentiation in fission yeast.

**Figure 1:**
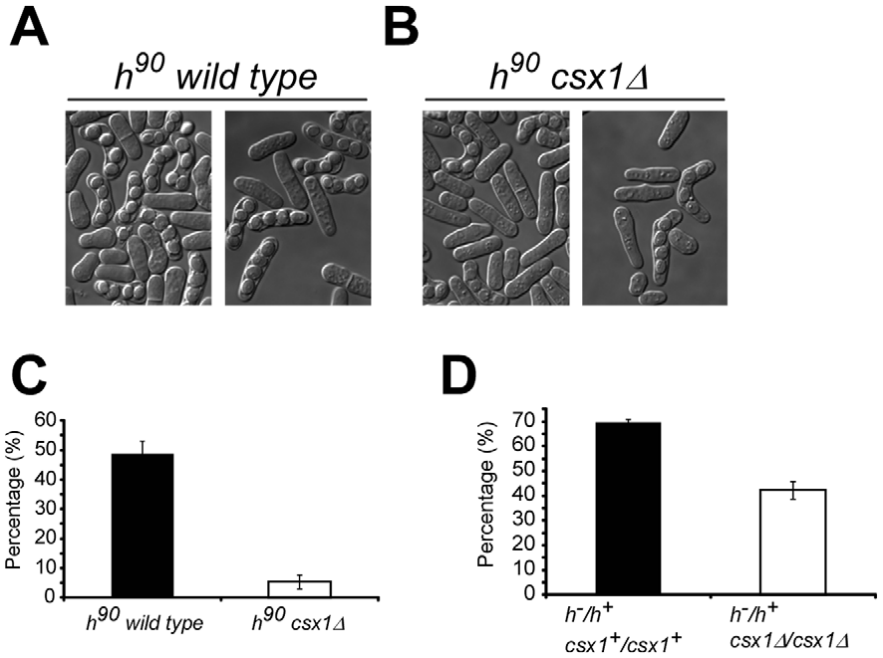
Csx1 is required for sexual differentiation in fission yeast. Morphology of homothallic wild type (A) and *csx1*Δ (B) strains after 48 hours in ME at 24°C. Pictures were taken using Nomarski filter. C. Mating percentage (conjugation/meiosis) reached by homothallic wild type and *csx1*Δ strains after 48 hours in ME at 24°C. Bars indicate standard error. D. Meiotic percentage reached by diploid h^−^/h^+^ *csx1^+^*/*csx1^+^* and h^−^/h^+^ *csx1*Δ/*csx1*Δ strains after 48 hours in ME at 24°C. Bars indicate standard error.

To analyze the ability to mate of cells lacking Csx1, we observed sporulation in homothallic strains, *h^90^* wild type and *h^90^ csx1*Δ. Both strains were plated in mating-inducing conditions (ME media) and incubated at 24°C for two days (Figure 1A, 1B).

In these experiments, we did not observe any morphological difference between tetrads formed in *csx1*Δ and wild type strains. However, the number of zygotes and tetrads in cells lacking Csx1 appeared to be much lower than in wild type.

To quantify mating efficiency we inoculated these strains in ME media. After 48 hours incubation at 24°C, the number of vegetative cells, zygotes and tetrads was measured, and the mating and sporulation ratio determined.

As it is shown in Figure 1C, about 45–55% of wild type cells mated in these conditions, while in *csx1*Δ cells, this ratio ranged between 4–8%. This result confirms that Csx1 is required for efficient sexual differentiation.

To elucidate whether mating defect in *csx1*Δ strain was due to a problem in conjugation and/or in meiosis, we performed a similar experiment using diploid strains. Diploid strains undergo meiosis under nitrogen starvation conditions, and they do not require previous conjugation.

Using the same media and temperature conditions employed with haploid strains, we quantified the sporulation ratio in *h^−^/h^+^ csx1^+^/csx1^+^* strain, used as reference, and in *h^−^/h^+^ csx1*Δ/*csx1*Δ strain. The sporulation ratio observed in *h^−^/h^+^ csx1*Δ/*csx1*Δ strain was about half of that reached by *h^−^/h^+^ csx1^+^/csx1^+^* strain (Figure 1D). This result indicates that diploid cells lacking Csx1 cannot undergo meiosis with the same efficiency as wild type strain, demonstrating that Csx1 may be required for meiosis. Csx1 deficiency has a stronger impact in haploid mating/sporulation than in diploids. This result indicates that Csx1 is also required for steps previous to meiosis, like nutrient sensing or conjugation.

### 2. Role of Csx1 in G1 cell cycle arrest

When homothallic strains are subjected to nitrogen starvation conditions they mate and sporulate, and one of the first steps in this response is the arrest of cell cycle at G1. Cells deficient in G1 cell cycle arrest (like *spc1*Δ and *atf1*Δ) are sterile [13].

**Figure 2:**
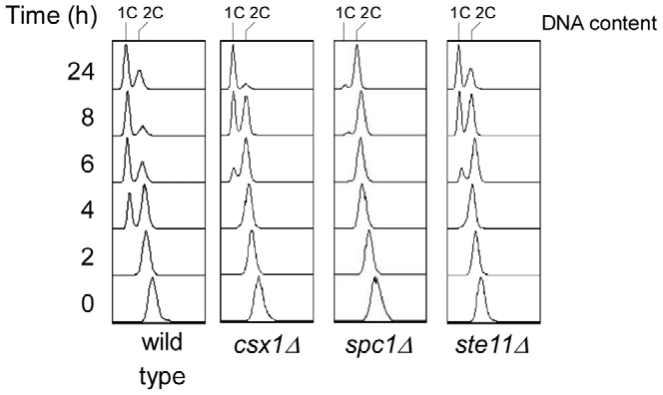
G1 arrest upon nitrogen starvation in several strains. Homothallic wild type, *spc1*Δ, *ste11*Δ, *csx1*Δ strains were incubated in EMM-N for up to 24 hours and samples were taken at the indicated times. DNA relative amount was estimated by the fluorescent signal amount emitted by propidium iodide and the flow citometry histograms represented.

Therefore, a possible explanation for *csx1*Δ sterility could be that homothallic *csx1*Δ strain did not arrest cell cycle at this stage. To test this possibility we performed flow cytometry assays after induction of sexual differentiation, analyzing DNA content of wild type and *csx1*Δ strains. We included *ste11*Δ and *spc1*Δ strains in the experiments, to compare with other well-known sterile strains (Figure 2). Cells were incubated in minimal media (EMM) and mating was induced in minimal media without nitrogen source (EMM-N) at 24°C, and they were harvested every two hours during a time course.

Wild type strains display an efficient G1 arrest as early as 4 hours after nitrogen depletion, and at eight hours most cells are arrested at G1 (Figure 2).

The profile displayed by *csx1*Δ and *ste11*Δ strains is almost identical, both mutant strains can arrest cell cycle at G1, but the arrest in these strains takes longer than in wild type strain, appearing the 1C peak after about 6–8 hours. In agreement with previous reports, *spc1*Δ strain profile demonstrates that Spc1 is required for cell cycle arrest at G1 [13].

By flow cytometry we confirmed that *h^90^ csx1*Δ strain did not show any evident defect in cell cycle, arresting at G1 after 8 hours in nitrogen starvation. We found that cells lacking Ste11 are able to arrest cell cycle at G1 stage and exhibit a similar pattern to cells lacking Csx1. This result is consistent with a role of Csx1 and Ste11 in similar mating processes or a regulation of one of them by the other.

### 3. Effect of *csx1*Δ in posttranscriptional regulation under nitrogen starvation conditions

Csx1 is an RNA binding protein that regulates *atf1^+^* stability under hydrogen peroxide treatment and is phosphorylated in a Spc1-dependent manner [21].

We hypothesized that the genetic relationship between Csx1 and Spc1/Atf1 might not be restricted to oxidative stress conditions, but also to sexual differentiation. Spc1 regulates the transcription factor Atf1 that, in turn, stimulates the production of *ste11^+^* mRNA under nitrogen starvation conditions. On the other hand, as we showed before, deficiencies in Csx1 or Ste11 cause similar defects in the mating process under nitrogen starvation conditions. We hypothesized that Csx1 could be playing a regulatory role in this process.

**Figure 3:**
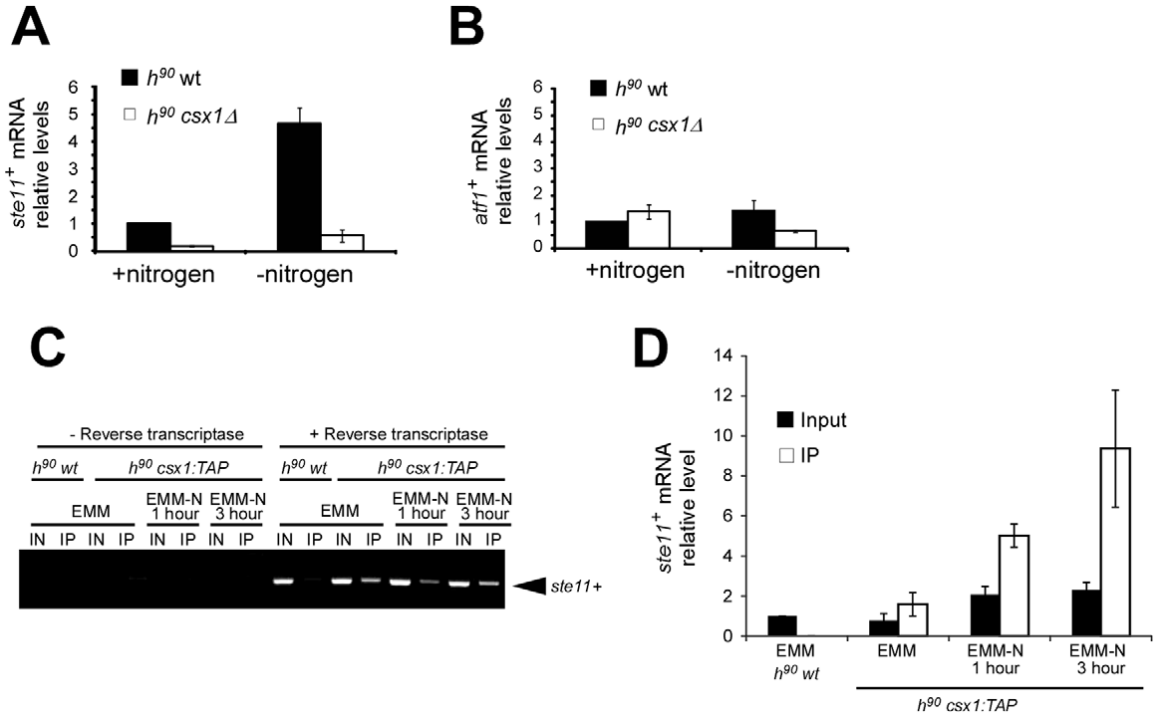
Csx1 binds *ste11^+^* mRNA and regulates its abundance. Quantitative real-time PCR analysis of *ste11^+^* mRNA (A) and *atf1^+^* mRNA (B) in homothallic wild type and *csx1*Δ strains in minimal media (EMM) and in the absence of nitrogen source (EMM-N) for 6 hours. Bars indicate standard error. C. Binding of Csx1 to *ste11^+^* mRNA. Cell extract was obtained (IN) and TAP immunoprecipitation (IP) was performed with homothallic wild type and *csx1:TAP* strains in minimal media (EMM) and in the absence of ammonium source (EMM-N) for 1 hour and 3 hours. RNA was isolated and cDNA generated by reverse transcription. *ste11^+^* mRNA was amplified by PCR and monitored by agarose electrophoresis (455 bps). D. Strains and treatment conditions were identical to those described in C. Binding of Csx1:TAP to *ste11^+^* mRNA was measured by reverse transcription followed by quantitative PCR. Actin mRNA was used as control. The graph represents *ste11^+^* mRNA relative levels in input samples and after Csx1:TAP purification (IP).

To test whether posttranscriptional response was altered in *csx1*Δ strain, we performed quantitative PCR to determine *atf1^+^* and *ste11^+^* mRNA levels under nitrogen starvation. We used *h^90^* wild type strain as reference (Figure 3A and 3B).

In wild type cells, *ste11^+^* mRNA is induced 4–5 times upon nitrogen starvation (Figure 3A). We noticed that in *h^90^ csx1*Δ strain, *ste11^+^* mRNA was reduced to 10% of wild type levels, both in regular minimal media and in nitrogen starvation conditions. Therefore, the induction of *ste11^+^* mRNA is strongly dependent on Csx1 activity, even in the presence of nitrogen source in the media.

*ste11^+^* mRNA transcription is induced by Atf1 and *atf1^+^* mRNA is a known target of Csx1 under hydrogen peroxide treatment [13], [21]. We, therefore, monitored the levels of *atf1^+^* mRNA in strains *h^90^* wild type and *h^90^ csx1*Δ (Figure 3B). The abundance of *atf1^+^* mRNA in *csx1*Δ and wild type strains after 6 hours in nitrogen starvation at 24°C was similar to the one observed in basal conditions. We therefore concluded that *ste11^+^* regulation was mediated by Csx1, independently of the effect exerted on *atf1^+^* mRNA.

### 4. Csx1 associates with *ste11^+^* mRNA

As shown in figure 3A, *ste11^+^* mRNA levels are altered in *h^90^ csx1*Δ strain. One possible explanation for this defect could be that Csx1 stabilized *ste11^+^* mRNA through protein-RNA interaction. To test this hypothesis we performed RNA-immunoprecipitation (RIp) assays using *h^90^ csx1:TAP* strain, using *h^90^* wild type strain as negative control of TAP purification. RIp assays were followed by reverse transcription (RT) reaction to generate cDNA. This cDNA was used as template in a PCR reaction to amplify *ste11^+^* mRNA.

We performed the same experiment in minimal media (EMM) and in minimal media without nitrogen (EMM-N) at 24°C, harvesting cells at 1 and 3 hours after nitrogen starvation (Figure 3C).

As was expected, in the negative control of RT reaction, where RNase-free water instead of reverse transcriptase was added, there was no amplification of *ste11^+^* mRNA. We can observe *ste11^+^* amplification in the IP extracts of *h^90^ csx1:TAP* strain, meaning that Csx1 is binding *ste11^+^* mRNA and this binding is maintained in all conditions described before. This result was also confirmed by reverse transcription followed by quantitative PCR (RT-qPCR) (Figure 3D).

Through these assays we confirmed that Csx1 is binding *ste11^+^* mRNA in the presence or absence of nitrogen. Furthermore, the binding of Csx1 to *ste11^+^* mRNA correlates with *ste11^+^* mRNA abundance. We have previously shown that Csx1 regulated Atf1 abundance through direct binding [21]. Therefore, our current working model indicates that Csx1 regulates *ste11^+^* mRNA stability through direct binding or association to the same ribonucleoprotein complex.

### 5. Ste11 rescues *csx1*Δ sterile phenotype

We have observed that strains lacking Csx1 are sterile. This disability in sexual differentiation in *csx1*Δ strain seems to be mainly due to *ste11^+^* mRNA abundance reduction and subsequent inappropriate response to nitrogen starvation conditions. To challenge this hypothesis, we monitored the effect of Ste11 ectopic expression in Csx1-deficient cells. We transformed homothallic *csx1*Δ strain with pREP1-Ste11. This plasmid contains *ste11^+^* ORF under *nmt1* promoter, repressible by thiamine. Using this plasmid we tested if overexpression of Ste11 in cells lacking Csx1 could rescue *csx1*Δ strain defects in sexual differentiation.

**Figure 4:**
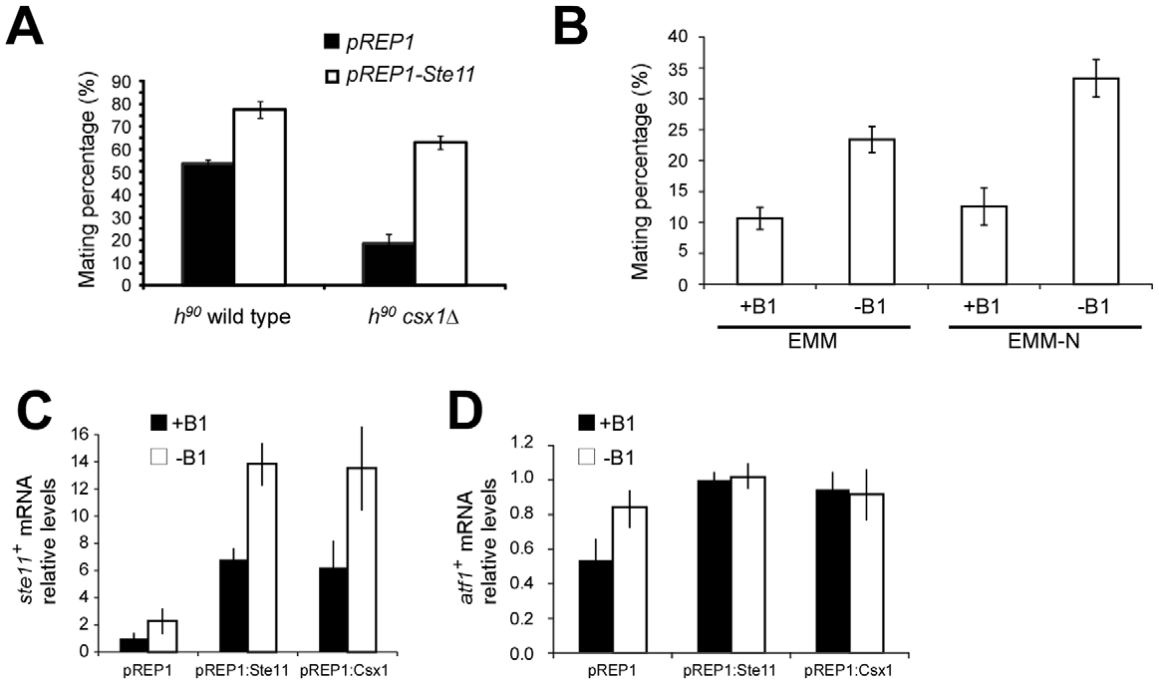
Overproduction of s*te11^+^* mRNA rescues *csx1*Δ sterility. A. Homothallic wild type and *csx1*Δ strains transformed with pREP1 and pREP1-Ste11 plasmids were plated in minimal media without nitrogen or thiamine and incubated for 48 hours at 24°C. Afterwards mating percentage was calculated. Bars indicate standard error. B. Homothallic *csx1*Δ strain was transformed with pREP1:Csx1 plasmid and mating percentage measured in minimal media with/without nitrogen source or promoter repressing thiamine (B1). In C and D, homothallic *csx1*Δ strains overexpressing Ste11 or Csx1, were grown in EMM-N media for 6 hours with or without thiamine (+B1/−B1) and *ste11^+^* mRNA (C) or of *atf1^+^* mRNA (D) measured by RT-qPCR.

This experiment was carried out in plates containing minimal media without nitrogen in the absence of thiamine to induce sexual differentiation and promoter activation, respectively. Homothallic wild type and *csx1*Δ strains were also transformed with control pREP1 plasmid (Figure 4A).

As was expected, in *h^90^* wt+pREP1 and *h^90^ csx1*Δ+pREP1 we observed similar mating effects of Csx1 on mating efficiency.

Overproduction of Ste11 induced an increase in mating efficiency in wild type strains (Figure 4A). In *h^90^ csx1*Δ+pREP1-Ste11 strain, under *nmt1^+^* promoter activation and therefore *ste11^+^* overexpression, we observed that mating percentage reached wild type levels. This result indicates that the mating defect in *csx1*Δ strains is mostly due to the downregulation of Ste11.

The overproduction of Csx1 in Csx1-deficient cells is able to rescue the mating capacity to levels similar to those found in wild type cells (Figure 4B). This increase in the mating efficiency correlates with increased levels of *ste11^+^* mRNA (Figure 4C). Levels of *atf1^+^* mRNA were analyzed in the same samples but we did not observe any significant changes (Figure 4D). These results are consistent with a model where Csx1 regulates *ste11^+^* mRNA levels that, consequently, affect mating efficiency in fission yeast.

## Discussion

Here, we have presented the RNA binding protein Csx1 as a novel regulator of sexual differentiation in fission yeast. The absence of Csx1 reduces mating capacity of homothallic *S. pombe* cells. This sterility can be fully rescued by overproduction of the transcription factor Ste11. Csx1 and *ste11^+^* mRNA co-precipitate, indicating that either Csx1 directly binds *ste11^+^* mRNA or they are part of the same multimolecular complex. The steady-state levels of *ste11^+^* mRNA are dependent on Csx1 activity, and cells deficient in Csx1 have reduced *ste11^+^* mRNA. We have not been able to measure *ste11^+^* mRNA half-life in response to Csx1 activity due to the low amount of *ste11^+^* mRNA in *csx1*Δ mutants. However, we have previously reported that Csx1 plays an important role on *atf1^+^* mRNA stabilization under oxidative stress [21]. Our working hypothesis proposes that Csx1 would be stabilizing *ste11^+^* mRNA, even before nitrogen starvation signals are triggered. However, further work should be done in order to demonstrate this important point.

In *S. pombe*, other RNA binding proteins have been demonstrated to play important roles in sexual differentiation either positively like Mei2 [3], or negatively like Msa1 and Msa2/Nrd1 [16], [17], [18]. All of them perform different biochemical functions and are regulated by different signals. Therefore, Csx1 positively regulate Ste11 that, in turn, regulates Mei2 [2].

The signals that allow Csx1 to distinguish the different stimuli and therefore associate with each mRNA remain unknown, but it is an interesting research field. One possible candidate for this coordination is the MAPK Spc1/Sty1 which, like Csx1, also participates in the response to oxidative stress and sexual differentiation in *S. pombe*.

Interestingly, there are several proteins in fission yeast that are important for both, mating efficiency and oxidative stress response. Proteins like Prr1, Spc1/Sty1 and Atf1 regulate *ste11^+^* mRNA levels and also the survival under several conditions of stress [12], [13]. This dual role points toward a coordination between both processes that should also be studied in other eukaryotes.

## Materials and Methods

### Strains and growth conditions

*csx1^+^* gene was deleted and TAP-tagged using a one-step PCR method [22] in a *h^90^* background using the following oligonucleotides:

**Csx1_delF**:TGACTTTTGTGTCTCATTGAAACTTTGTTGTTCATTCATATTACTTACTTTCTTTTACTTTTTTTTGGATATCTATTTAACGGATCCCCGGGTTAATTAA;

**Csx1_delR**:AATAAAAAAAATCACGAGAGCACCCTTCAGTTCTTTAAGACATTAAACTAACTTGATCAGGAGCCCTCGAAAACTTATACGAATTCGAGCTCGTTTAAAC;

**Csx1_tagF**:GCTTGCCTCCTCGTTCTTATTCTACATTTAATTGTACTGGTCAATACTTGCAACCTTCTCTACGCTTGTCACGCGATTCACGGATCCCCGGGTTAATTAA.

pREP1 plasmids were introduced into *S. pombe* following lithium acetate protocol [23]. For overexpression experiments using the *nmt1* promoter, cells were plated in EMM without NH_4_Cl (nitrogen source) and without thiamine, and incubated at 24°C for 48 hours. To induce mating, *S. pombe* strains were cultivated in Edinburgh minimal media (EMM), washed and resuspended in EMM without NH_4_Cl (EMM-N) [24]. To calculate mating percentage, fresh cultures were plated in ME (Malt Extract) media for 48 hours at 24°C. 10 µl of a cell dilution were placed into Neubauer chamber and tetrads, zygotes and normal cells were quantified, and the ratio of mating calculated. For calculations of haploid mating, we performed the following operation: (2xA+2xB)/(2xA+2xB+C), where A is the number of zygotes, B the number of tetrads and C the number of vegetative cells.

Pictures were taken after 48 hours incubation in ME media at 24°C using Nomarski optics.

### Quantitative PCR

Total RNA was extracted as described in Lyne et al. [25]. cDNA was generated using Reverse Transcription System kit (Promega®), following manufacturer's guidelines. To amplify *ste11^+^*, *atf1^+^* and *act1^+^* (actin) mRNAs the following primers were used:

**187.Ste11_5_probe:** ACCTAAAACCCCGAATACCG;

**188.Ste11_3_probe:** TTAGAATTGGGCAACCAAGG;

**185.Atf1_5:** AACCCCTACTGGAGCTGGAT;

**186.Atf1_3:** GGGAACCTGGGAGAGTAAGC;

**189.Act1_5_probe:** AGCACCCTTGCTTGTTGACT;

**190.Act1_3_probe:** CTCATGAATACCGGCGTTTT.

Actin was used as endogenous control. Quantitative analyses were performed using the ΔΔCt method.

### RNA immunoprecipitation

RNA immunoprecipitation was performed using homothallic wild type and *csx1:TAP* strains. Immunoprecipitation was carried out in a rotational wheel at 4°C using Pan IgG mouse magnetic beads (Dynabeads®-Invitrogen) as described by Amorim and Mata [26]. Specific oligonucleotides were used for Ste11 detection by PCR:

**187.Ste11_5_probe:** ACCTAAAACCCCGAATACCG

**188.Ste11_3_probe:** TTAGAATTGGGCAACCAAGG

### Flow cytometry

Flow cytometry was used to estimate the relative DNA content of fission yeast cells, and define the cell cycle stage of the population. Time course was performed growing all strains in EMM and samples were taken at 2, 4, 6, 8 and 24 hours after mating induction (nitrogen starvation). 3 ml of cells were harvested at 0.3 OD_600_. 7 ml of ethanol were added and incubated at 4°C for 10 minutes.

Cells were spin down and resuspended in 1 ml of PBS-Triton-HCl solution (1 ml PBS, 0.5% Triton-X-100, 0.1 N HCl) and incubated for 10 minutes at room temperature.

Cells were spin down and resuspended in RNAse A at 250 mg/ml. After 2-hour incubation at 37°C, 200 µl of cells were mixed with 800 µl of PBS. That mixture was gently sonicated to separate cellular aggregates and propidium iodide at 2.5 µg/ml was added to each sample. The flow cytometer used in this study was FACSCalibur (Bencton Dickinson).
